# Cryptococcal Pneumonia in a Patient on Tyrosine Kinase Inhibitor Therapy: How Common Is It?

**DOI:** 10.7759/cureus.47884

**Published:** 2023-10-28

**Authors:** Deny Sung, Shubani Singh, Sanjeev K Goswami

**Affiliations:** 1 Internal Medicine, St. Joseph’s Medical Center, Stockton, USA; 2 Pulmonary and Critical Care Medicine, St. Joseph’s Medical Center, Stockton, USA

**Keywords:** immunocompromised, immunotherapy, ibrutinib therapy, chronic lymphocytic leukemia, cryptococcal pneumonia, cryptococcus gattii

## Abstract

Cryptococcal pneumonia is identified as a fungal infection of the lungs, with *Cryptococcus neoformans* and *Cryptococcus gattii *as the most common culprits. *Cryptococcus neoformans* primarily affects immunocompromised individuals while *Cryptococcus gattii* infections occur mostly in immunocompetent hosts. We present a 76-year-old male on ibrutinib due to a history of chronic lymphocytic leukemia who had multiple hospitalizations for pneumonia and was later diagnosed with cryptococcal pneumonia through positive bronchoalveolar lavage fungal culture and lymph node biopsy.

## Introduction

*Cryptococcus (C.) neoformans* and *Cryptococcus gattii* are the two species that cause the majority of cryptococcal infections in humans [1]. A 10-year population-based register of cases in Australian patients demonstrated that *C. neoformans* infections occurred primarily in immunocompromised hosts while *C. gattii* infections occurred mostly in immunocompetent hosts [2]. There have been increasing reports of *C. gattii* infections in patients on immunotherapy [3]. The specific effect of immunotherapy can range from favorable to detrimental depending on factors relating a host to an organism [3]. Fungal infections with cryptococcal isolates have a low index of clinical suspicion among physicians, thus posing a diagnostic challenge for cryptococcosis in such hosts.

## Case presentation

The patient is a 76-year-old male who presented to the emergency department due to shortness of breath for one week with an associated cough productive of blood-streaked sputum, pleuritic chest pain, and subjective fevers. This patient had a history of hypertension, chronic obstructive pulmonary disease (COPD) without supplemental oxygen requirement, and chronic lymphocytic leukemia (CLL) on ibrutinib therapy. The ongoing symptoms started soon after discharge from a recent three-day hospitalization for COPD exacerbation and left lower lobe pneumonia that was treated with steroids and antibiotics. Bronchoscopy or cultures were not obtained at that time since the patient improved with empiric therapy. Our patient did not have a personal history of tuberculosis or valley fever but did endorse a distant history of cigarette use 30 years prior. Additional review of systems was unremarkable.

**Figure 1:**
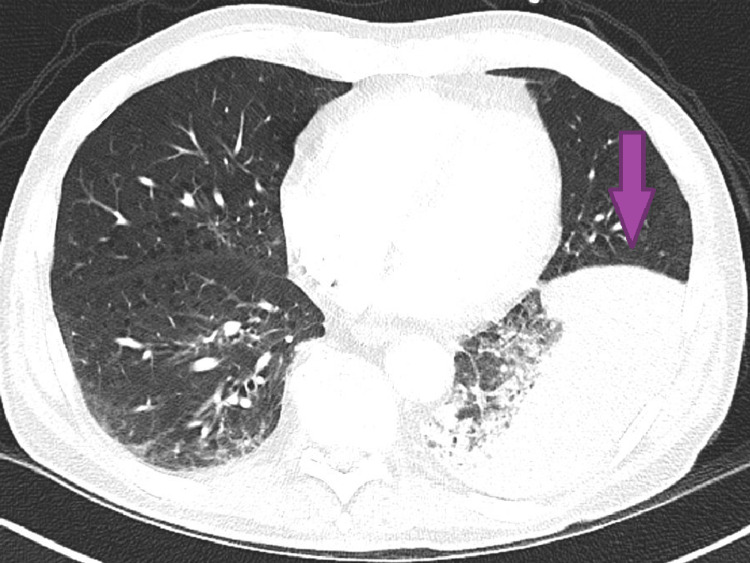
Axial view of chest CTA demonstrating left lower lobe consolidation CTA: computed tomography angiography

**Figure 2:**
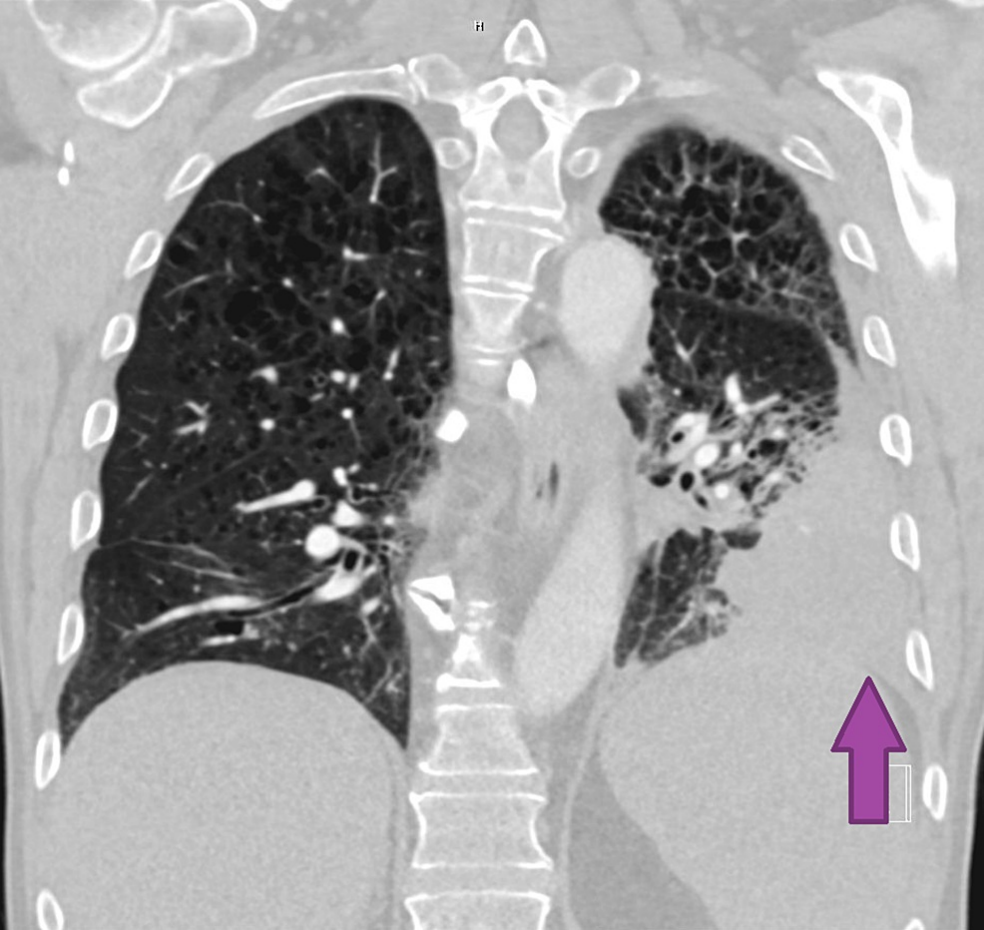
Coronal view of chest CTA showing left lower lobe consolidation CTA: computed tomography angiography

Vitals were unremarkable, and he was on 2 liters per minute of oxygen via nasal cannula for comfort. Physical exam was only significant for decreased breath sounds in the left lower zone. Chest X-ray was suspicious for a large mass in the left lower lobe, a moderately sized left pleural effusion, and unspecified chronic changes in the right lower lobe. Computed tomography angiography (CTA) of the chest showed significant worsening of the left lower lobe consolidation, severe emphysema, and mediastinal and hilar lymphadenopathy but was negative for pulmonary embolism (Figures 1, 2).

Chest ultrasound excluded bilateral effusions and was unremarkable. Serologies for COVID-19, Legionella, influenza A and B, Mycoplasma, and Coccidioides were negative. Remarkable lab results included an erythrocyte sedimentation rate of 45 and a C-reactive protein level of 11.61. Despite a normal leukocyte count, the patient was initiated on piperacillin-tazobactam, vancomycin, doxycycline, and fluconazole for empiric broad-spectrum coverage.

Further evaluation with bronchoscopy, endobronchial ultrasound with lymph node transbronchial needle aspiration biopsies, and bronchoalveolar lavage fungal cultures revealed *Cryptococcus gattii *in all the samples. Antimicrobial therapy was de-escalated per culture sensitivity results, and the patient was discharged on doxycycline and fluconazole with close pulmonology outpatient follow-up.

## Discussion

Cryptococcal infections are usually acquired through the inhalation of desiccated yeast cells or spores and can not only cause an infection of the lungs but can also affect the central nervous system in severe, disseminated cases [1]. This case joins a growing body of reports detailing cryptococcal pneumonia in patients on Ibrutinib therapy. Increased awareness of the possibility of cryptococcal pneumonia as a differential diagnosis is important for early detection and appropriate treatment. According to a study of invasive fungal infections from France, the incidence of cryptococcosis was about 5.2% [4]. In patients with CLL, cryptococcal infections can occur in the first two to six months of treatment with Ibrutinib [3]. This medication is Bruton’s tyrosine kinase inhibitor, which is often used to treat CLL.

*Cryptococcus* spp. are opportunistic yeast infections, but *C. gattii* is known to cause severe infections in immunocompetent patients [1]. A small study of 378 patients in 2018 suggested that the use of ibrutinib could be a risk factor for developing serious infections, including cryptococcal pneumonia. Although this study was not able to definitively show ibrutinib as an inciting factor for infections, it showed that patients on ibrutinib still developed fungal infections without the classic risk factors for fungal infections [5]. A potential explanation for this association was demonstrated by a study in X-linked immunodeficient mice with a deficiency of B cells and a reduction of natural immunoglobulin M (IgM) due to a mutation in the Bruton’s tyrosine kinase (Btk) gene. These mice had a significantly higher brain fungal burden with *Cryptococcus neoformans* following a pulmonary infection, suggesting that an explicit threshold of IgM or B cell subsets is required to prevent disseminated cryptococcosis [6]. This research lends insight, albeit limited, to the association between ibrutinib and *C. gattii*; however, targeted research exploring this unique species is required to draw distinct conclusions.

## Conclusions

We present a patient who developed cryptococcal pneumonia while on ibrutinib therapy for CLL. The exact relationship between ibrutinib and cryptococcosis is unclear, but it could stem from the inhibition of Bruton’s tyrosine kinase and its effects on IgM or B cell levels. Further understanding of the mechanism of action of Ibrutinib and the possibility of such immunotherapy as a risk factor for cryptococcosis would facilitate early diagnostic efforts for patients with similar presentations.
